# Being right matters: Model-compliant events in predictive processing

**DOI:** 10.1371/journal.pone.0218311

**Published:** 2019-06-13

**Authors:** Daniel S. Kluger, Laura Quante, Axel Kohler, Ricarda I. Schubotz

**Affiliations:** 1 Department of Psychology, University of Muenster, Muenster, Germany; 2 Otto-Creutzfeldt-Center for Cognitive and Behavioral Neuroscience, University of Muenster, Muenster, Germany; 3 Goethe Research Academy for Early Career Researchers, University of Frankfurt, Frankfurt, Germany; 4 Department of Neurology, University Hospital Cologne, Cologne, Germany; Queen Mary University of London, UNITED KINGDOM

## Abstract

While prediction errors (PE) have been established to drive learning through adaptation of internal models, the role of model-compliant events in predictive processing is less clear. Checkpoints (CP) were recently introduced as points in time where expected sensory input resolved ambiguity regarding the validity of the internal model. Conceivably, these events serve as on-line reference points for model evaluation, particularly in uncertain contexts. Evidence from fMRI has shown functional similarities of CP and PE to be independent of event-related surprise, raising the important question of how these event classes relate to one another. Consequently, the aim of the present study was to characterise the functional relationship of checkpoints and prediction errors in a serial pattern detection task using electroencephalography (EEG). Specifically, we first hypothesised a joint P3b component of both event classes to index recourse to the internal model (compared to non-informative standards, STD). Second, we assumed the mismatch signal of PE to be reflected in an N400 component when compared to CP. Event-related findings supported these hypotheses. We suggest that while model *adaptation* is instigated by prediction errors, checkpoints are similarly used for model *evaluation*. Intriguingly, behavioural subgroup analyses showed that the exploitation of potentially informative reference points may depend on initial cue learning: Strict reliance on cue-based predictions may result in less attentive processing of these reference points, thus impeding upregulation of response gain that would prompt flexible model adaptation. Overall, present results highlight the role of checkpoints as model-compliant, informative reference points and stimulate important research questions about their processing as function of learning und uncertainty.

## Introduction

Predicting upcoming events constitutes one of the fundamental qualities of brain function. Based on internal models shaped by previous experience, top-down predictions are compared to bottom-up sensory signals [1]. Redundant components of perceptual information are disregarded whereas surprising expectancy violations are propagated upward in the processing hierarchy [2–3]. Model adaptation in consequence of such prediction errors (PE) has been proposed to be the foundation of associative learning mechanisms [4–5], as unexpected events are particularly informative with regard to their current context. Importantly, probabilistically occurring *expected* events have also been suggested to inform the internal model [6]: While PE instigate model adaptation, expected events verify model-based predictions. These verifications are particularly informative when we face uncertain environments. A recent fMRI study [7] found that in uncertain environments, so-called *checkpoints* (CP) emerged as points in time where distinctive processing of expected events pointed to a context-sensitive adaptation in predictive processing. While the entire stimulus sequence could be predicted reliably in stable environments, unstable environments prompted stepwise predictions. This way, CP were used to verify the internal model in order to predict the next section accordingly. Thus, while model *adaptation* is induced by prediction errors, context-dependent model *evaluation* does not seem to require expectancy violations. Instead, selected time points carry information about the on-line validity of the internal model, raising the intriguing question of how checkpoints and prediction errors functionally relate to one another.

For the present study, we employed the paradigm from [7] in an electroencephalography (EEG) experiment. Exploiting the temporal benefits of EEG, we aimed to further understand the functional relationship of CP and PE as well as their respective evolution over time. Specifically, we aimed to show how functional commonalities of and central distinctions between the two event types translate to electrophysiological signals.

Participants performed a serial pattern detection task in which they were asked to press and hold a response button whenever they detected a short or long ordered digit sequence (e.g. 1-2-3-4-5, length of either 5 or 7 items) within an otherwise pseudorandom stream of coloured single digits. Expectable sequence length was cued by digit colour and occasionally violated by premature terminations or unexpected extensions. In addition to these two types of prediction errors, checkpoints were defined as sequential positions where PE could potentially occur, *but did not*. Thus, although checkpoints were exclusively sampled from regular events consistent with the previous cue, their occurrence was probabilistically modulated by blockwise manipulation of *irreducible uncertainty*. Irreducible uncertainty refers to uncertainty that cannot be reduced further and remains even after successful (i.e., ideal) learning [8–9]. Going back to our research question, both checkpoints and prediction errors provide central information for model evaluation or adaptation, respectively, whereas deterministic standard trials (STD) did neither. Consequently, we first hypothesised a joint event-related (ERP) component of CP and PE (compared to STD) reflecting recourse to the internal model. The P3b component has been conclusively shown to co-vary with subjective improbability or unexpectedness of a stimulus [10–12]. Such highly informative events supposedly initiate contextual updating [13–14] or memory-based revision of mental representations [15]. Importantly, the P3b is elicited by behaviourally relevant rather than merely deviant stimuli in order to facilitate motor responses [16–17], making it a promising candidate for a joint physiological component of checkpoints and prediction errors.

Aside from the aforementioned conceptual commonalities of CP and PE, one critical distinction remains, namely the mismatch signal that is intrinsic to prediction errors. N400 is elicited by a multitude of sensory events and its amplitude is known to scale with event surprise in language [18–19], recognition memory (reviewed in [20]), and arithmetic tasks [21–22], presumably marking modality-independent integration of incongruous information. More generally, N400 has been discussed as a modality-independent index of representations needing revision in light of expectancy violations (for review, see [11]). Consequently, we hypothesised an enhanced N400 component for highly surprising, model-incongruent PE to reflect this mismatch signal in contrast to expectation-compliant CP: While information from both event types has to be integrated into existing model structures, the excess effort of integrating prediction error information should result in a more pronounced N400 component.

Complementing ERP analyses, we assessed topographic microstates [23] for a multivariate, assumption-free comparison of the temporal dynamics underlying CP and PE processing. This way, we aimed to characterise the two event classes using similarities and differences in the onset, duration, and strength of their respective network activation.

Finally, we employed performance-based subgroup analyses of behavioural data to further assess the implications of statistical learning on individual reaction time patterns. Specifically, we hypothesised strong reliance on cue information to induce both beneficial and maladaptive responses towards CP and PE, respectively.

## Materials and methods

The study was conducted according to the principles expressed in the declaration of Helsinki and approved by the Local Ethics Committee of the University of Münster (Department of Psychology).

### Participants

A total of 32 neurologically healthy, right-handed volunteers (26 female) at the age of 23.4 ± 2.5 years (M ± SD) participated in the study for payment or course credit. Participants were recruited from the university’s volunteer database and had (corrected-to-) normal vision. Written informed consent was obtained from all participants prior to the start of experimental procedures. One participant was excluded from further data analysis due to poor behavioural performance during the experiment; a second participant was excluded due to technical difficulties during the EEG session. Therefore, all reported analyses are based on a sample of 30 participants (25 female, age 23.2 ± 2.5 years).

### Stimulus material

**Fig 1:**
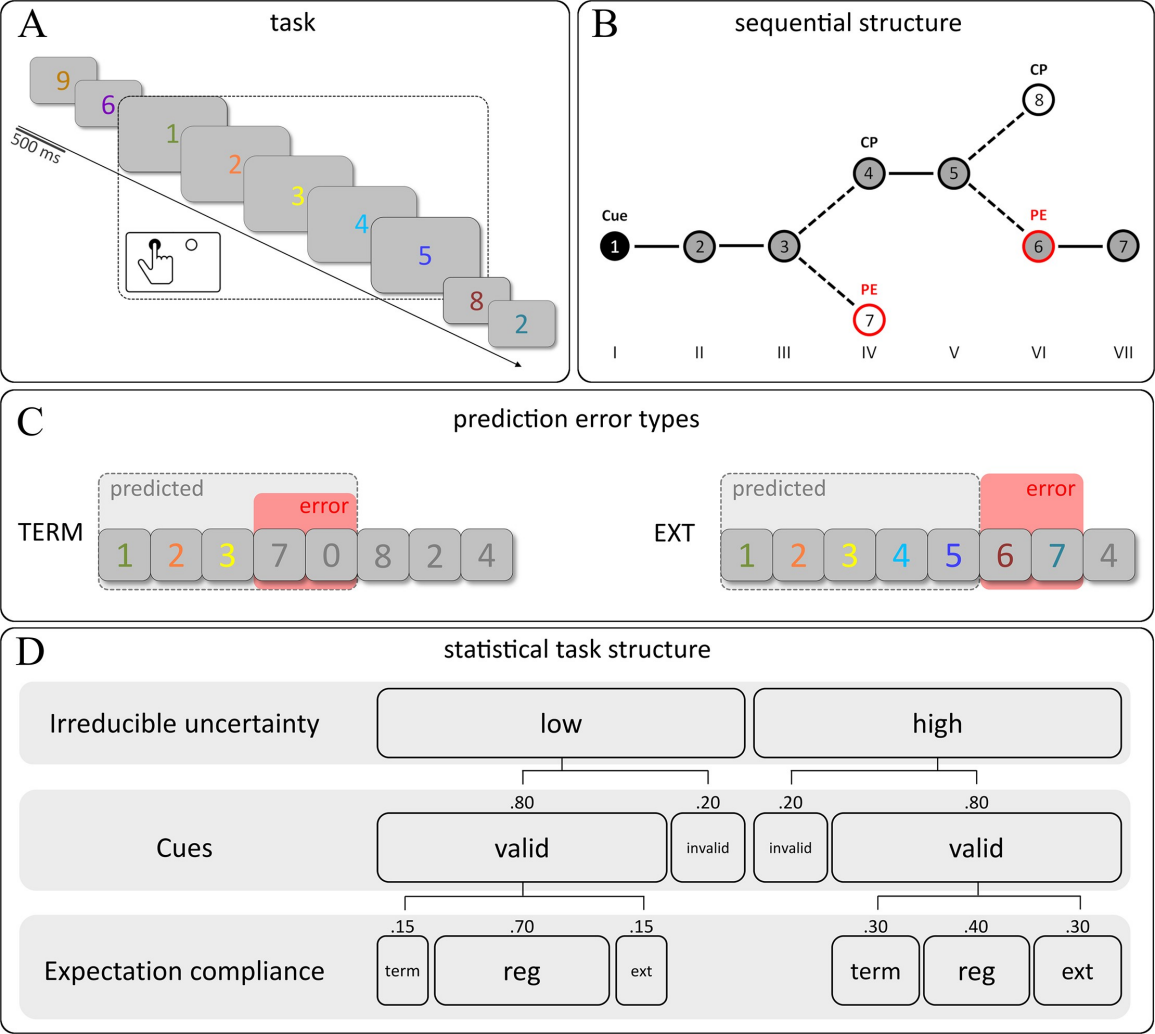
(A) Exemplary trial succession and time frame of the corresponding response for ordered sequences. Sequential trials have been highlighted for illustrative purposes. (B) Schematic structure of a short ordered sequence showing the positions of checkpoints (CP) and prediction errors (PE, red). At the fourth position, the sequence could either be terminated (PE) or continued as expected (CP). Similarly, the sixth position contained either the regular end (CP) or an unexpected extension of the sequence (PE). (C) Cue-based expected sequence length and resulting prediction errors for terminated and extended short ordered sequences (*expectation compliance*). (D) Local transition probabilities for terminated, regular, and extended sequences depending on the respective level of irreducible uncertainty.

Task and stimulus material of the present study were adopted from a previous fMRI study conducted in our lab [7]. In short, participants were shown pseudorandomly coloured single digits presented for 500 ms in the centre of a light grey computer screen (see Fig 1A). Presentation frequencies for all colours and digits were equally distributed both within and across blocks of approximately 6 minutes. Each block contained *ordered sequences* increasing the previous digit by one (e.g. 1–2–3–4–5; Fig 1A, left) embedded in *random trials* with no discernible relation between consecutive digits. In order to balance sequential starting points across digits, the ascending regularity necessarily included the 0 character and continued in a circular fashion after the figure 9 (e.g. 8–9–0–1–2).

Undisclosed to the participants, two colours were exclusively used as cues (fixed validity *p* = .80) to indicate the start of ordered sequences: one colour marked the first digit of a *short* ordered sequence (regular length of five digits), a second colour marked the first digit of a *long* ordered sequence (regular length of seven digits). Each participant was assigned two individual cue colours from distinct hues.

Prediction errors, i.e. violations of cue-based expectations with regard to sequence length, were induced by manipulation of the sequences’ *expectation compliance*. While the cues indicated the length of *regular* ordered sequences (e.g. seven digits for long ordered sequences), *terminated* sequences were shortened by two items. Conversely, *extended* sequences were prolonged by two items (Fig 1C). In addition to prediction errors, *checkpoints* were defined as events of interest for subsequent analyses. In line with the design of our previous study [7], checkpoints were sampled from positions of *potential* terminations and extensions, when expected stimuli were in fact presented (see Fig 1B). Finally, the composition of regular, terminated, and extended sequences within a particular block was varied across blocks. This way, the *irreducible uncertainty* of a block was set to be either *low* or *high* (Fig 1D). Low uncertainty blocks could be seen as statistically stable regarding cue-based expectations whereas highly uncertain blocks formed a more unstable statistical structure. The experiment was programmed and run using the Presentation 14.9 software (Neurobehavioral Systems, San Francisco, CA, USA).

### Task

Participants were instructed to press and hold the left button of a response box with their right index finger as soon as they noticed an ordered sequence. Release of the button was to indicate the end of the ordered sequence.

### Experimental procedures

The study was conducted on two consecutive days. On the first day, participants completed a training session to familiarise themselves with the task and to provide them with implicit knowledge of the cues and the underlying statistical structure of the experiment. The training consisted of two blocks (one block of low and high uncertainty, respectively) with a total duration of approximately 12 minutes. Importantly, at no point during the training or the EEG session was it revealed that there was informational content in some of the colours (i.e. the cues) or that the blocks varied in their respective statistical structure (i.e. their level of uncertainty).

The second day included the EEG session as well as a subsequent post-measurement. The EEG session consisted of eight blocks (four blocks of each uncertainty level) with a total duration of approximately 48 minutes. Detailed information on trial numbers per block and condition is provided in Supporting Information (S1 Table). Participants were sitting comfortably on a chair in a darkened, sound-dampened and electrically shielded EEG booth. They were instructed to avoid blinking the best they could, most importantly during button presses. Experimental procedure and task during the EEG session were otherwise identical to the training session.

Following the EEG session, participants completed a behavioural post-measurement in order to assess their implicit knowledge of the cue information. To this end, they were shown one final experimental block (duration approx. 5 min) on a computer outside the EEG booth, performing the identical task as before. Crucially, only half of the ordered sequences were cued by the colours learned during the training and the EEG session. The other half began with fixed but different colours that had indeed been presented during training and EEG, but not as cues for the respective participant. Therefore, these colours were non-informative in that they contained no implicitly learned information concerning upcoming trials. In a verbal interview following the post-measurement, all participants denied having noticed any colour-related regularity.

### Behavioural data analysis

Statistical analyses of behavioural responses were performed in R (R Foundation for Statistical Computing, Vienna, Austria). First, correct and incorrect responses were aggregated separately for training, EEG session, and post-measurement for each participant. Incorrect responses were further divided into misses (no response over the course of a sequence) and false alarms (response occurring without presentation of sequential trials). Participants’ overall performances were assessed via the discrimination index PR [24].

Reaction times for button presses and releases were assessed for the EEG session and post-measurement. Onset latency was calculated as reaction time relative to the onset of the second trial of any particular ordered sequence (i.e. the earliest possible point to detect a sequential pattern). Offset latency was calculated as reaction time relative to the onset of the first random trial after a particular sequence. Reaction times occurring either before the cue trial (i.e. earlier than -500 ms) or more than 2000 ms after the end of the sequence were excluded.

We used a repeated-measures analysis of variance (ANOVA) to assess potential differences in offset latency as a function of expectation compliance and uncertainty during the EEG session. Furthermore, effects of cue learning on onset latency during the post-measurement were assessed by means of a paired *t*-test (learned vs new cue colours). Finally, a data-driven subgroup analysis based on participants’ post-test performance was conducted to assess differential effects of stimulus surprise on response patterns as well as premature and anticipatory button releases as a function of cue learning. Where appropriate, results of paired *t*-tests were corrected for multiple comparisons at *p* = .05 using the false discovery rate (fdr) correction [25].

### Single-trial analyses: Event-specific surprise

In addition to global context effects of uncertainty, single-trial behavioural and physiological correlates of CP and PE conceivably depend on how much information is carried by respective stimuli. For single-trial analyses of reaction times and ERPs, we modelled event-specific surprise following the notion of an ideal Bayesian observer (see [26]). Surprise *I(x*_*i*_*)* was defined as the improbability of event *x*_*i*_, i.e.

$$I(x_{i})=-\ln\;p(x_{i})$$

with

$$p(x_{i})=\frac{n_{j}^{i}+1}{\sum_{k}n_{k}^{i}+1}$$

where $n_{j}^{i}$ denotes the total number of occurrences of outcome *j* (*terminated*, *regular*, *extended*) up to the current observation *i* relative to the sum of all past observations (with *k* for all possible outcomes).

### EEG data analysis

#### EEG data acquisition and data preprocessing

Scalp EEG was recorded from 62 Ag/AgCl-electrodes mounted in a BrainCap TMS electrode cap (Brain Products, Gilching, Germany) using the BrainVision Recorder software (Brain Products, Gilching, Germany). All scalp channels were measured against a ground electrode at position FPz and referenced against FCz during recording. Two additional electrooculogram (EOG) electrodes were applied above and below the right eye for the detection of horizontal and vertical eye movements. All impedances were kept below 10 kOhm. EEG was recorded at a sampling rate of 1 kHz with recording filters set to 0.1–1000 Hz bandpass.

EEG preprocessing was conducted in EEGLAB [27]. Data segments containing experimental breaks were discarded prior to independent component analysis (ICA). Resulting components distinctly reflecting eye movements were subsequently rejected manually (mean = 2.83 components) using the SASICA toolbox [28]. Data were then filtered with a 0.1 Hz low cut and 30 Hz high cut filter and recalculated to common average reference. Based on participants’ overall pattern of reaction times at the end of sequences, a time frame of [–100, 600] ms was defined for the analysis of event-related potentials (ERP) and multivariate segmentation. Epochs containing artefacts were discarded by semiautomatic inspection with an allowed maximum/minimum amplitude of ± 200 μV and voltage steps no higher than 50 μV per sampling. Channels with a high proportion of outliers (kurtosis criterion: z > 6) were replaced by a linear interpolation of their neighbour electrodes (M = 1.8 interpolated channels).

#### Event-related potentials

Averages of the epochs representing our events of interest were calculated separately for each participant. Prediction errors were defined as violations of cue-based expectations of sequence length. For terminations, the event onset was time-locked to the first unexpected *random* digit, whereas extensions were defined with the onset time-locked to the first unexpected *sequential* digit. *Checkpoints* were defined as positions of potential expectation violations when an expected stimulus was in fact presented. At these points in time, based on a previous study [7], we hypothesised an incoming regular (i.e., expected) stimulus to be checked for either a termination (i.e. a check occurring during the ongoing sequence) or an extension (i.e. a check at the regular end) of the ordered sequence. Due to their unambiguous characteristic and temporal distinctiveness, digits at the fourth position of every long extended sequence were defined as *sequential standard trials*. The number of valid trials per condition (after artefact rejection) is summarised in Supporting Information (S2 Table). Finally, grand averages across participants were calculated for all events of interest.

Using the Mass Univariate ERP Toolbox [29], we employed a two-stage approach to assess reliable ERP differences between conditions: First, we restricted our analyses to specific time frames and electrodes for stringent testing of our hypotheses. In a second step, we performed a whole-brain analysis including all time points to increase sensitivity. In each case, ERPs from the respective conditions were submitted to a repeated measures cluster mass permutation test [30] using a family-wise significance level of α = .05. Repeated measures *t*-tests were performed for each comparison using the original data and 5000 random within-participant permutations of the data. For each permutation, all *t*-scores corresponding to uncorrected p-values of *p* = .05 or less were formed into clusters. The sum of the *t*-scores in each cluster defined the "mass" of that cluster and the most extreme cluster mass in each of the 5001 sets of tests was used to estimate the distribution of the null hypothesis.

To recap, ERP analyses were conducted to highlight functional commonalities of and distinctions between checkpoints and prediction errors. To this end, both CP and PE were first compared to non-informative standard trials (STD) within the P3b time window (300–600 ms). In line with our hypotheses, differential correlates of prediction errors and checkpoints were finally assessed in a direct comparison within the N400 time frame (300–500 ms).

#### Multivariate segmentation

We used the Cartool software package (available via www.sites.google.com/site/cartoolcommunity) for a segmentation of event-related EEG data sets into topographic maps. This procedure was first introduced by Lehmann and colleagues [23] to describe what they termed *functional microstates*, meaning brief time periods of stability in otherwise fluctuating field configurations. More generally, this segmentation allows the assessment of spatial field characteristics and their temporal dynamics (see [31]. As these topographic ERP analyses consider information from all electrodes (i.e. the field configuration as a whole), they offer a multivariate approach to investigating effects between time periods or experimental conditions without a priori selection.

The methodology behind topographic ERP analyses has been described in great detail (see [32] for an excellent step-by-step tutorial) and is briefly outlined here. Based on the so-called AAHC (*atomize and agglomerate hierarchical clustering*) algorithm, Cartool first iteratively generated clusters of ERP topographies to identify template maps separately for each experimental condition. A cross-validation criterion was then used to determine which number of template maps optimally described the group-averaged ERPs (i.e. how many template maps were needed to maximise explained variance in the data). Finally, the optimal number of cluster maps per experimental condition was fitted back to the original data, allowing us to compare onset and duration of the same template maps across conditions.

Multivariate segmentation analysis was conducted to assess the number and topographic distribution of template maps underlying ERPs of checkpoints, prediction errors, and standard trials as well as differences in their timing and/or field power across conditions.

## Results

### Behavioural results

#### EEG session

**Fig 2:**
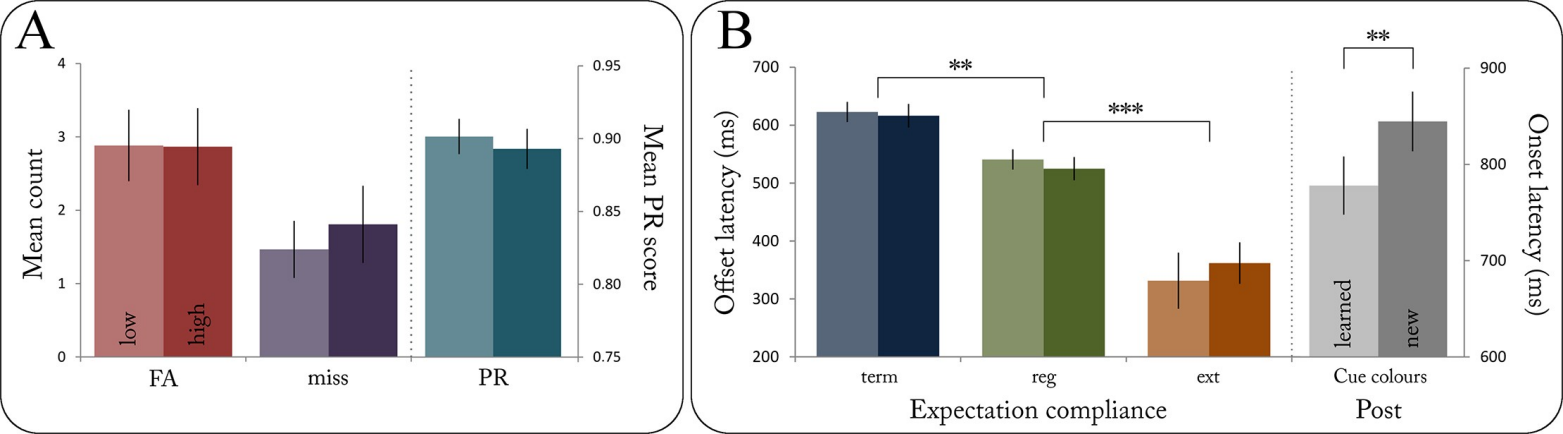
(A) Mean count of false alarms (FA) and misses per block as well as mean PR score as a function of uncertainty. (B) Mean offset latencies for terminated, regular, and extended sequences as well as mean onset latencies for learned and new cue colours during post-measurement. ** = *p* < .01, *** = *p* < .001.

All participants showed an overall high level of performance with a mean PR score of *M*_*PR*_ = 0.90 (*SD* = 0.06) during the EEG session, indicating good attentiveness throughout the experiment. Mean PR scores did not differ significantly between experimental blocks (*F*(7, 248) = 0.03, *p* = .999) or as a function of block uncertainty (*t*(29) = 1.58, *p* = .139, see Fig 2A).

The repeated-measures ANOVA yielded a significant main effect of expectation compliance on offset latency (*F*(2, 58) = 51.54, *p* < .001). Post-hoc pairwise *t*-tests revealed participants’ button releases to be significantly slower after terminated (M = 619.48 ms, SD = 96.55 ms) than after regular (M = 532.81 ms, SD = 99.74 ms, fdr-adjusted *p* = .003) as well as after extended sequences (M = 346.68 ms, SD = 219.35 ms, fdr-adjusted *p* < .001). The difference between extended and regular sequences was significant as well (fdr-adjusted *p* < .001, see Fig 2B). This pattern of offset latencies fully replicated the findings from our previous fMRI study (see [7]). Neither the main effect of uncertainty (*F*(1, 29) = 0.05, *p* = .821) nor the interaction term of uncertainty X expectation compliance (*F*(2, 58) = 1.72, *p* = .187) reached statistical significance, suggesting that participants were able to discriminate regular from manipulated sequences regardless of the respective uncertainty level. The number of misses (*t*(29) = -1.89, *p* = .068) and false alarms (*t*(29) = 0.10, *p* = .923) did not differ significantly between high and low uncertainty blocks (see Fig 2A).

#### Post-measurement

Participants performed equally well during the post-measurement (*M*_*PR*_ = .90, *SD* = 0.05) as they had during the EEG session. The post-measurement was conducted in order to assess cue learning: If participants had learned the association of cue colours and prospective ordered sequences over the course of the training and the EEG session, they could be expected to respond more quickly to sequences beginning with established cue colours than to those starting with new colours during the post-measurement. Indeed, the corresponding *t*-test confirmed a significant difference between learned and new cue colours (*t*(29) = -2.47, *p* = .01, one-tailed): Participants exhibited a shorter reaction time to learned cue colours (M = 788.02 ms, SD = 168.00 ms) than to new cue colours just introduced during the post-measurement (M = 844.59 ms, SD = 172.31 ms; see Fig 2B).

#### Performance-based subgroup analyses

**Fig 3:**
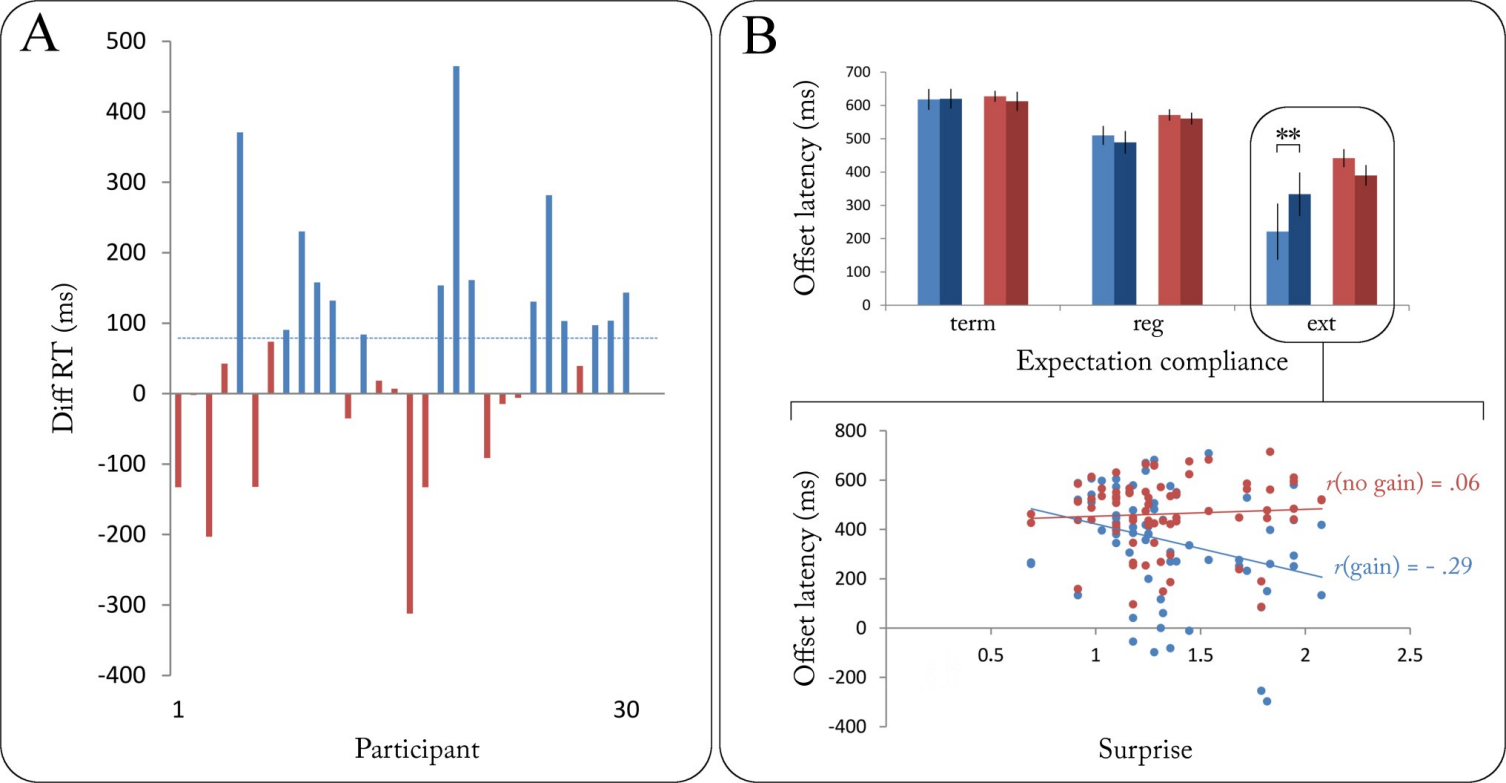
(A) Individual gains in reaction time (defined as the difference in reaction time following new minus learned cues) during post-measurement. Positive values indicate quicker button presses following learned cues. Blue dotted line depicts Mdn_Diff_ = 78.70 ms. Participants were consequently median-split into a *gain* group (blue) and a *no gain* group (red). (B) Upper panel: Mean offset latencies as a function of expectation compliance for gain (blue) and no gain group (red). Significant differences only shown for high vs low uncertainty for the sake of clarity (see Fig 2B for differences between levels of expectation compliance). Lower panel: Correlations between offset latency and trial-specific surprise value of sequential extensions for both groups. ** = *p* < .01.

Based on participants’ reaction times at onset during the post-measurement, the sample was median-split into two equal groups (*n* = 15): The first group had shown a gain in response speed following the learned cue colours (*gain*), whereas the second group had not (*no gain*, see Fig 3A). The gain group showed a significantly higher difference between reactions to new and learned cue colours (M = 178.54 ms, SD = 106.62 ms) than did the no gain group (M = -45.40 ms, SD = 82.24 ms, *t*(26.64) = 6.29, *p* < .001, one-sided).

The rationale behind comparing the two subgroups’ behavioural performance was that a stronger association of cue colour and sequence length (as reflected by a pronounced gain in response speed during post-measurement) should entail distinct response patterns at the end of regular and manipulated sequences. We repeated the offset latency ANOVA separately for gain and no gain groups and found the overall main effect of expectation compliance to be present in both groups (gain: *F*(2, 28) = 30.15, *p* < .001; no gain: *F*(2, 28) = 28.23, *p* < .001; see Fig 3B). Notably, only the gain group showed a significant interaction of expectation compliance and irreducible uncertainty (*F*(2, 28) = 7.98, *p* = .002): Button releases at the end of extended sequences occurred significantly earlier when uncertainty was low (M = 221.16 ms, SD = 328.87 ms) than when it was high (M = 333.75 ms, SD = 252.23 ms, *t*(14) = 2.90, *p* = .012).

By definition, extensions were on average more surprising under low uncertainty due to their low presentation rate in these blocks. Importantly, however, event-specific surprise values of extensions also fluctuated under high uncertainty (albeit to a lesser extent). Thus, the reported uncertainty effect on the gain group’s responses after extended sequences might be generalised across uncertainty levels in such a way that more surprising extensions–regardless of global contextual features–evoked shorter offset latencies in the gain group: If excess reliance on cue information had in fact determined the behavioural effect found for the gain group, these participants should have responded equally fast to locally surprising extensions irrespective of global uncertainty. Corroborating this hypothesis, we found a significant negative correlation of stimulus-bound surprise and offset latency after extended sequences for the gain group (*r*(72) = —.29, *p* = .013) but not for the no gain group (*r*(72) = .06, *p* = .617). The difference between the two correlation coefficients was found to be significant (*Z* = 2.11, *p* = .017, one-tailed).

**Fig 4:**
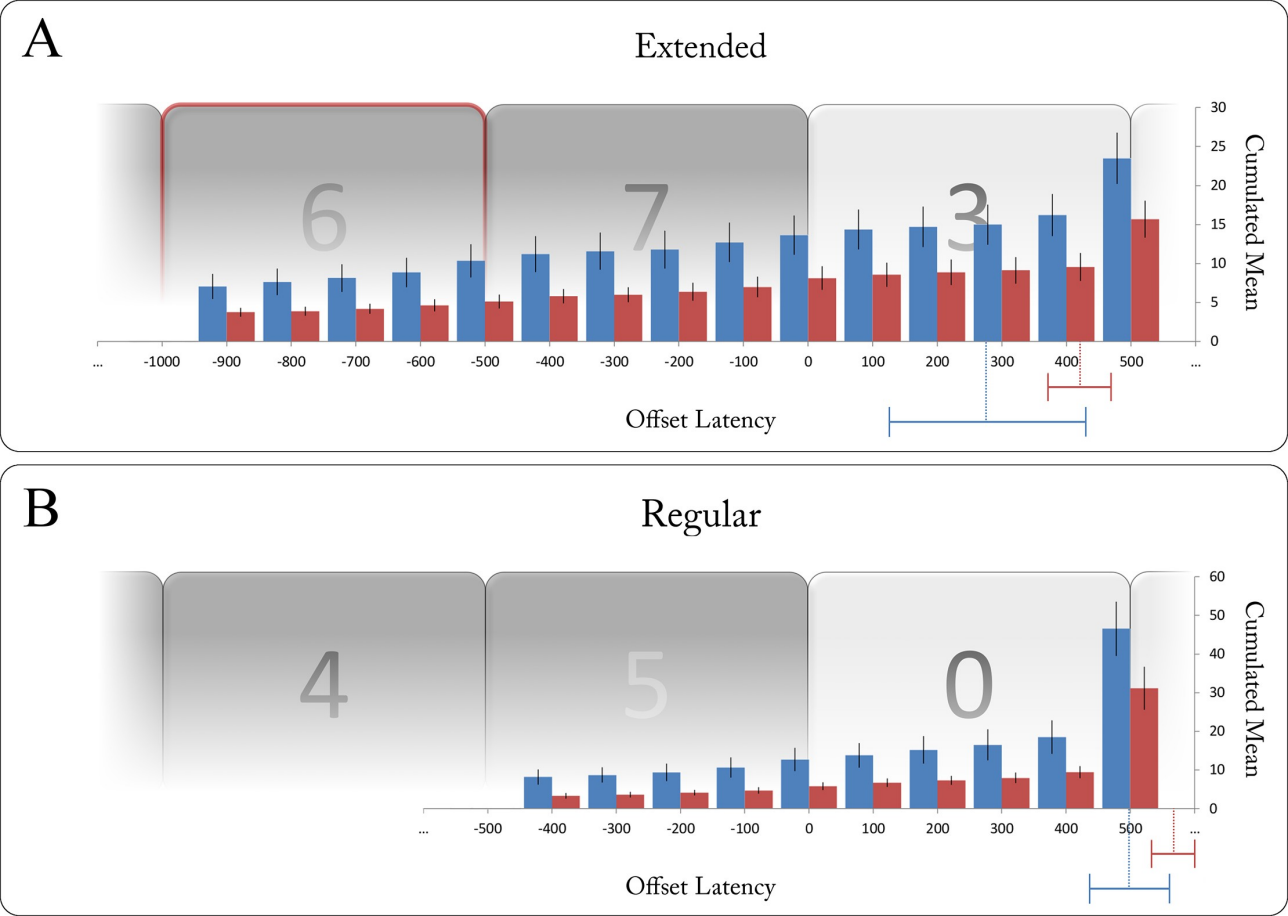
(A) Mean count of button releases during the experiment up to selected offset latencies for gain (blue) and no gain group (red). Shown here for an exemplary short extended sequence (length of 7 digits), the gain group was found to release the response button more frequently at offset latencies between -1000 and +500 ms (i.e. between the onset of the unexpected sequential digit [red frame] and the offset of the first non-sequential) following extended sequences. Dotted lines and bars depict mean offset latencies for regular sequences per group ± 2 SEM. (B) Similarly, shown here for a short regular sequence (length of 5 digits), the gain group was found to release the response button more frequently at offset latencies between -500 and +500 ms (i.e. between the onset of the last sequential digit and the offset of the first non-sequential digit) following regular sequences. Dotted lines and bars depict mean offset latencies for extended sequences per group ± 2 SEM.

An intuitive explanation for earlier releases following highly surprising extensions would be that the gain group not only released the button more quickly, but also more often prematurely: Conceivably, the more stable the cue information had been learned, the more likely would the response button be released at the expected sequence end rather than at the actual end. We assessed two additional questions with regard to more specific distinctions in behaviour: First, we hypothesised the gain group to more frequently respond prematurely to extended sequences, i.e. at the ‘would-be’ end of the sequence had it not been extended (see Fig 1B). Recall that unexpected extensions occurred at the sequential positions where–based on the cue information–a non-sequential digit was expected. Accordingly, as illustrated in Fig 4, we compared the two groups’ button releases within the interval of -1000 (onset of the unexpected sequential digit) and 500 ms (offset of the first non-sequential digit) around the end of extended sequences. Supporting our hypothesis, the gain group was found to have a significantly higher number of releases within the [–1000, 500] ms time frame than the no gain group (*t*(15) = 22.28, *p* < .001, one-sided; see Fig 4A). The group difference in incremental releases per 100 ms window was also found to be significant (*t*(15) = 2.35, *p* = .017, one-sided).

Second, stronger expectations by means of more accessible cue information within the gain group could conceivably lead to a similar pattern of early responses following regular sequences. The gain group could therefore be expected to more frequently release the response button within a brief interval of ± 500 ms around the end of regular sequences. Responses during the last sequential digit (i.e. offset latency between -500 and 0 ms) would reflect an anticipatory release of the response button whereas responses during the first non-sequential digit (0–500 ms) would reflect a quick detection of the sequence end. Both anticipatory and quick releases after the end of a regular sequence were hypothesised to be positively associated with the degree to which the colour-length association had been learned. Fittingly, the gain group was found to have a significantly higher number of releases within the ± 500 ms time frame than the no gain group (*t*(10) = 9.47, *p* < .001, one-sided; see Fig 4B). The group difference in incremental releases per 100 ms window showed a non-significant trend (*t*(10) = 1.81, *p* = .05, one-sided).

### EEG results

#### Event-related potentials

**Fig 5:**
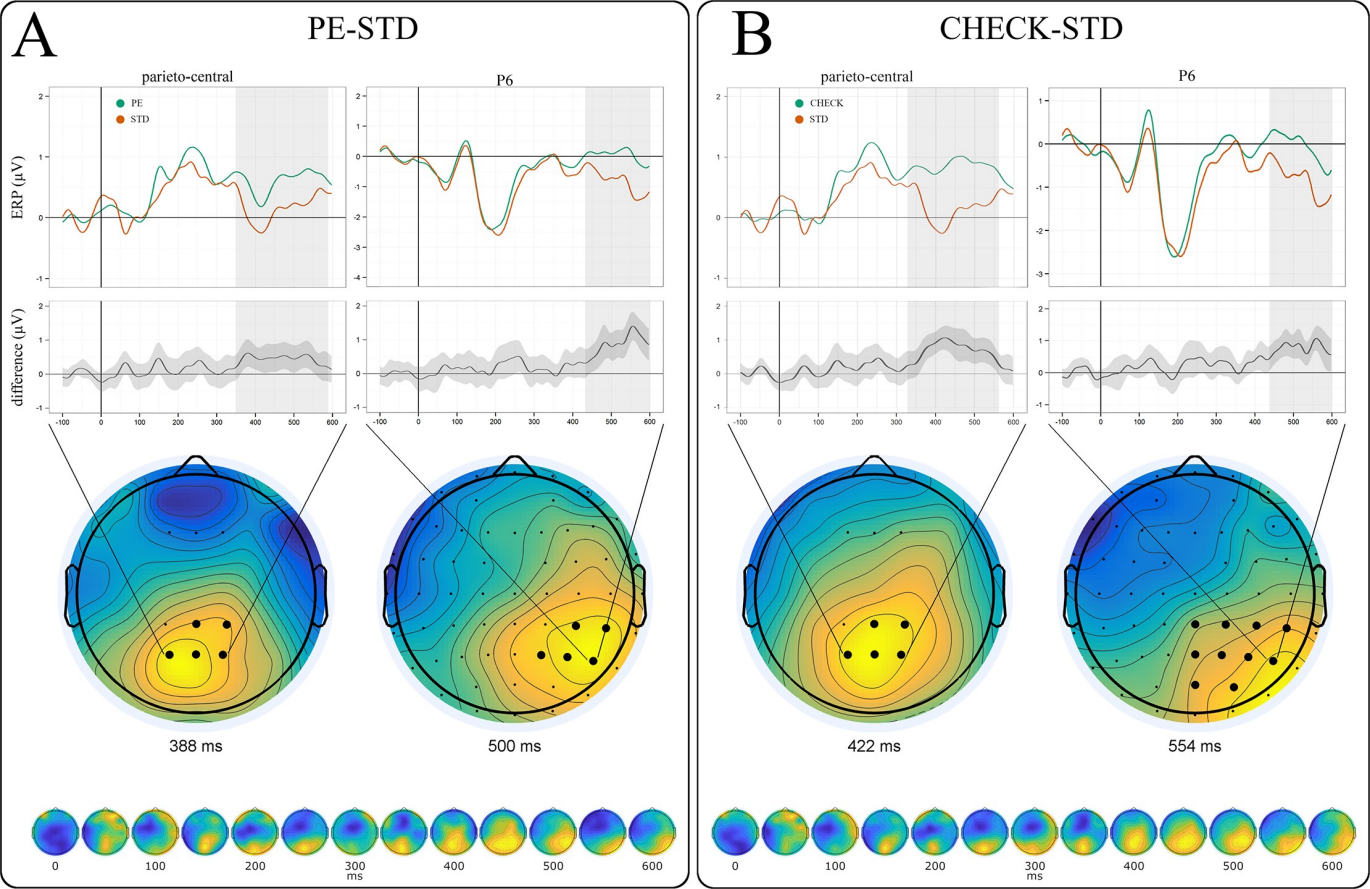
(A) Significant ERP differences between prediction errors and sequential standards included a parieto-central P3b (left) as well as a right-lateralised P600 component peaking over electrode P6 (right). P3b topography shows the frontal and parietal subsets of electrodes used for the analysis (bottom left). Significant clusters are marked in bold. (B) ERP differences between checkpoints and sequential standards were equally reflected in significant P3b (left) and P600 components (right). Respective bottom panels show component evolution over time (all electrodes, no temporal constraints).

Based on our hypotheses, we first tested prediction errors and sequential standards for reliable differences in the P300 time frame. We analysed all time points between 300 and 600 ms (1350 comparisons in total) from two subsets of electrodes: one parieto-central subset (CP1, CPz, CP2, P1, Pz, P2) to detect a posterior P3b component and a fronto-central subset (F1, Fz, F2) controlling for anterior P3a effects (see [15] for a review of P3a and P3b topographies). Supporting our hypothesis, we found a significant P3b over the parieto-central electrodes (352–576 ms) peaking around 388 ms (Fig 5A). No significant potentials were found in the fronto-central subset.

Subsequently, all time points between 0 and 600 ms were included in a two-sided whole-brain analysis to assess reliable differences exceeding our hypotheses (18600 comparisons in total). In addition to the reported P3b effect (see S1 Fig for comparison), we found a significant ERP component resembling a P600 with a right-lateralised parietal scalp distribution peaking around 500 ms (Fig 5A). While timing, scalp distribution, and the underlying experimental manipulation are fitting for a P600 component, the reported effect is caused at least in part by a more pronounced negativity of STD (instead of a PE-related positivity). We therefore refrain from interpreting this finding and suggest future studies specifically address P600 modulation as a function of local probabilities.

Like prediction errors, checkpoints are probabilistic, highly informative sequential positions with an immediate relevance for behaviour. Therefore, one would expect a certain degree of similarity between PE and checkpoint ERPs when compared with deterministic, behaviourally non-informative standard trials. ERPs from checkpoints and sequential standards were submitted to a one-sided analysis including all time points between 300 and 600 ms (1350 comparisons in total) and the two electrode clusters described above. The analysis revealed a pattern very similar to previous prediction error ERPs, including both a significant posterior P3b (324–574 ms, peaking around 422 ms) and a right-lateralised P600 (peaking around 554 ms, Fig 5B). Notably, P3b and P600 peak latencies thus occurred slightly earlier for prediction errors than for checkpoints. No significant potentials were found in the fronto-central subset.

**Fig 6:**
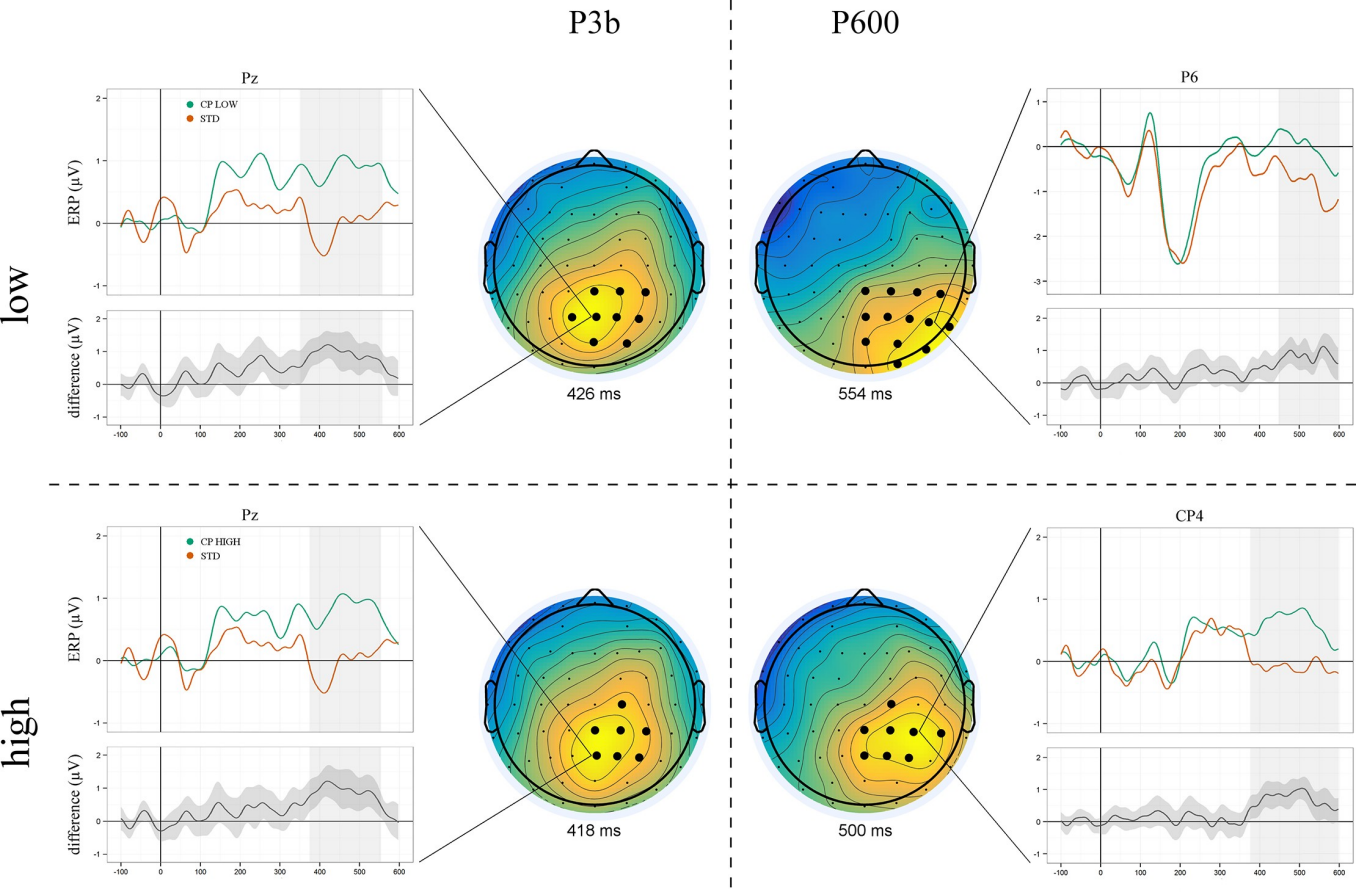
Grand averaged ERPs of low (top row) vs high uncertainty checkpoints (bottom row) and sequential standards. Checkpoints elicited significant P3b (left) and P600 components (right) irrespective of the uncertainty level. Note that, while uncertainty did not modulate P3b scalp distribution or peak latency, the P600 elicited by high uncertainty checkpoints showed an earlier peak and a slightly more frontally distributed topography.

Since strategic adaptation of CP processing as a function of context uncertainty was one of the central objectives of the previous fMRI study, we subsequently split the analysis to separately assess high and low uncertainty checkpoint ERPs. P3b and P600 were found for checkpoints in both uncertainty conditions (Fig 6). Interestingly, while the P3b component was virtually identical in both latency and scalp distribution, we found subtle differences regarding the P600: At the group level, the activation peak occurred ~50 ms earlier and slightly more frontally for high (500 ms at CP4) than for low uncertainty checkpoints (554 ms at P6, see Fig 6).

Recall that we observed an earlier P3b peak for prediction errors (388 ms) than for low (426 ms) and high uncertainty checkpoints (418 ms). In contrast, P600 peak latencies were identical for PE and high uncertainty CP (500 ms) and earlier than for low uncertainty CP (554 ms). This pattern of ERP results suggests a close functional relationship of prediction errors and (particularly high uncertainty) checkpoints (see Figs 5A and 6). This relationship and its variation under uncertainty were the main objective of our subsequent multivariate segmentation analysis (see below).

**Fig 7:**
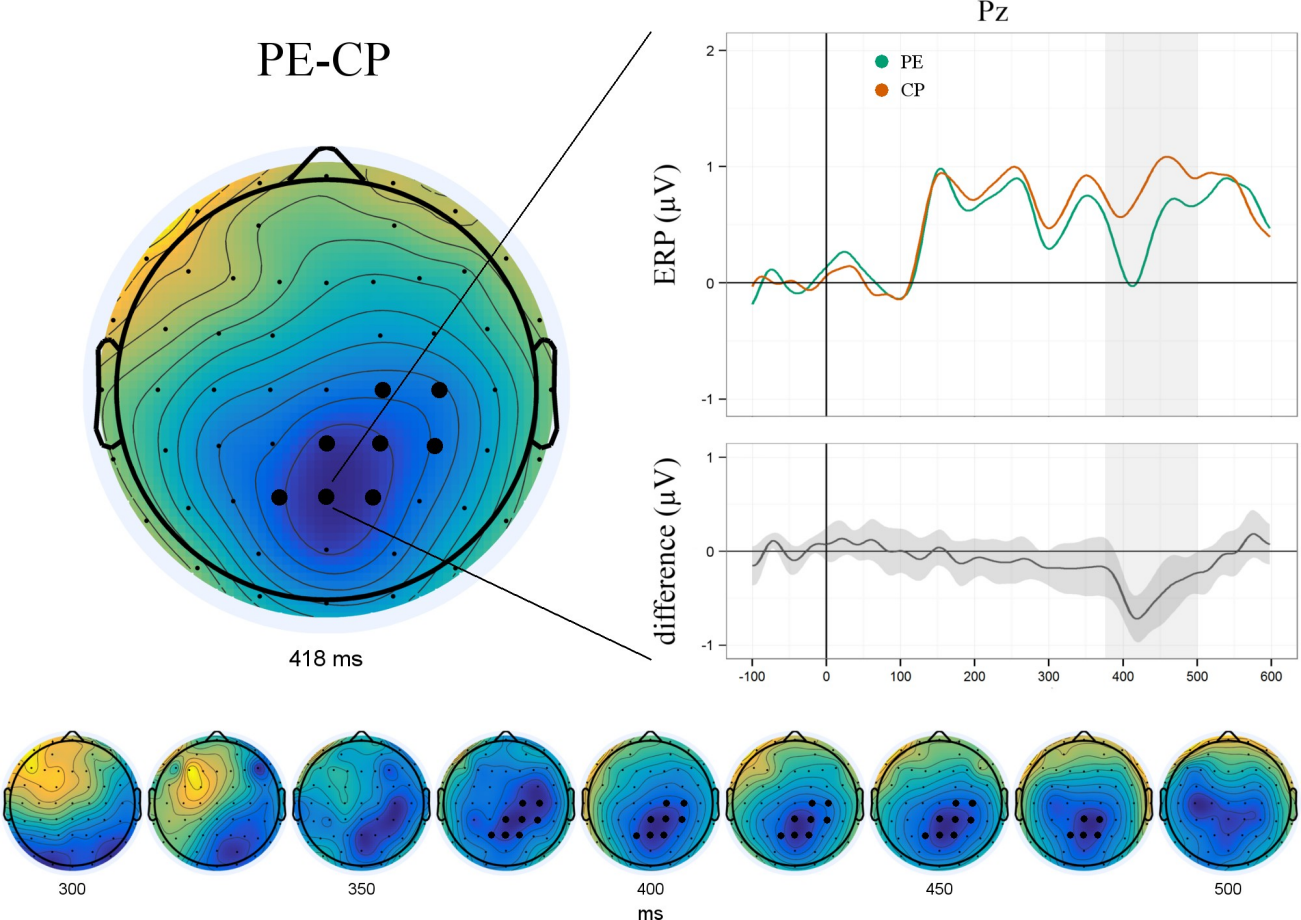
The direct comparison of prediction errors and checkpoints revealed a significant N400 component peaking around 418 ms over parieto-central electrodes. Bottom panel shows component evolution over time.

Given the reported conceptual and functional similarities between prediction errors and checkpoints, their direct contrast was meant to reveal the correlate of expectation violation definitive of PE. We hypothesised this mismatch to be reflected in an enhanced N400 component. Accordingly, we included all time points between 300 and 500 ms in a one-sided whole-brain analysis (6262 comparisons in total). PE were found to elicit a significantly enhanced N400 over parieto-central electrodes (338–500 ms) peaking around 418 ms (Fig 7). No additional significant components were found in the subsequent whole-brain analysis including all time points between 0 and 600 ms (18600 comparisons in total).

#### Multivariate segmentation

**Fig 8:**
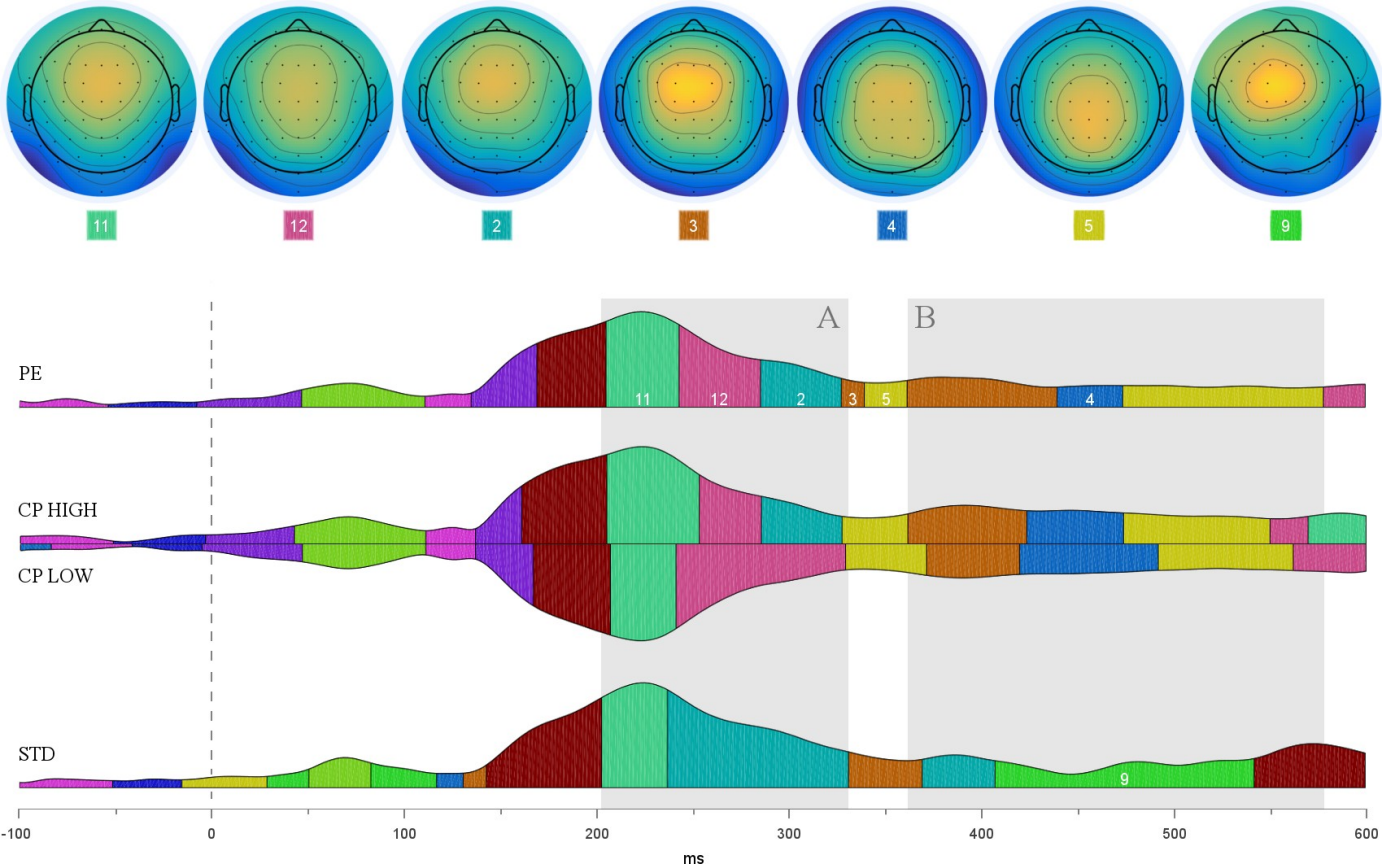
Global field power (GFP) of group-averaged ERPs for prediction errors, checkpoints under high/low uncertainty, and sequential standard trials time-locked to stimulus onset. Coloured segments within the area under the curve depict distinct topographic configurations (*template maps*, TM) as revealed by hierarchical clustering. Upper panel shows scalp distributions of TM depicted in Box A (TM 11, 12, 2) and B (TM 3, 4, 5, 9). Note that the CP LOW curve was flipped for illustrative purposes only and did not differ in polarity.

Cartool’s meta-criterion showed that group-averaged ERPs of checkpoints, prediction errors, and standard trials were optimally described by a set of 12 topographic template maps (TM). Fig 8 shows the temporal progressions of these topographies for each condition. Visual inspection suggested notable differences between conditions within two main time frames. First, following a virtually simultaneous onset of fronto-centrally distributed TM 11 (around 204 ms), PE and high uncertainty CP exhibited a sustained frontal cluster (TM 2, 284–326 ms) after transitioning through a more global TM 12 (Fig 8, Box A). Whereas this frontal shift was not found for low uncertainty CP, it was even more pronounced for STD (i.e., with a higher amplitude and an earlier onset). After fitting the group-level template maps onto individual subject data, one-sided *t*-tests confirmed a significantly greater global field power of TM2 for STD compared to PE (*t*(13.08) = 1.91, *p* = .039) and CP HIGH (*t*(14.83) = 1.83, *p* = .044). Similarly, onsets of TM2 occurred significantly earlier for STD than for CP HIGH (*t*(20.86) = - 1.95, *p* = .033). The comparison of STD and PE showed a non-significant trend (*t*(15.42) = - 1.67, *p* = .057).

Second, ERP time courses showed differential topographic as well as temporal configurations during a later time frame (starting at around 360 ms). Prediction errors and both checkpoint conditions shared a frontal-to-parietal shift (TM 3–5) with particular differences in cluster onset and duration (Fig 8, Box B). In contrast, sequential standard trials showed a distinct ongoing frontal topography with a slight dominance of left hemisphere sources (TM 9, 406–540 ms). Group-level onset and duration for the reported topographies are listed in Table 1.

**Table 1:** Group-level onset and duration of selected template maps for PE, high/low uncertainty checkpoints, and sequential standard trials. Time frame for grand average ERP analysis [–100, 600] ms.

TM class	Condition			
	*PE*	*CP HIGH*	*CP LOW*	*STD*
*TM 2*				
Onset (ms)	284	284	-	236 | 368
Duration (ms)	42	42	-	94 | 38
*TM 3*				
Onset (ms)	326 | 360	360	370	330
Duration (ms)	12 | 78	62	48	38
*TM 4*				
Onset (ms)	438	422	418	-
Duration (ms)	34	50	72	-
*TM 5*				
Onset (ms)	472	472	328 | 490	-
Duration (ms)	104	76	42 | 70	-
*TM 9*				
Onset (ms)	-	-	-	406
Duration (ms)	-	—	-	134
*TM 11*				
Onset (ms)	204	204	206	202
Duration (ms)	38	48	30	34
*TM 12*				
Onset (ms)	242	252	240	-
Duration (ms)	42	32	88	-

## Discussion

Predicting events of everyday life, our internal model of the world is constantly compared to sensory input we perceive. Prediction errors induced by unexpected events are deemed particularly informative in that they instigate learning through model updating. We show here that information is equally sampled from *expected* events at particularly relevant checkpoints, suggesting that under uncertainty, model-affirmative events similarly prompt recourse to the internal model. Both checkpoints and prediction errors showed a significant P3b component when compared to sequential standards, indexing the relative (im)probability of CP and PE occurrence. Conversely, the direct comparison of CP and PE revealed a significant N400 component as the mismatch correlate elicited solely by prediction errors. Combined with findings from behavioural and functional microstate analyses, checkpoint characteristics highlight the significance of informative reference points for abstract predictive processing, raising intriguing questions for future research.

### Functional characteristics of checkpoints

In order to establish a more precise characterisation of checkpoints, they have to be related to and dissociated from two other event types: First, since checkpoints are regular events, they share the expectedness of sensory input with sequential standards. In contrast to these standards, however, checkpoints are probabilistic and therefore informative with regard to task context and behavioural requirements. Second, PE are equally informative but do carry a mismatch signal that requires behavioural adaptation in opposition to the internal model.

As hypothesised, the significance of checkpoints and prediction errors as particularly meaningful points in time was reflected in a joint P3b component compared to least informative standards. Often discussed as an index of enhanced information transmission and allocation of resources [33–34], P3b is well suited to reflect exploitation of information at these sequential positions. More precisely, an incoming stimulus is evaluated in context of previous stimuli by comparing it to information from working memory [35–36]. Such monitoring is immediately beneficial for stimulus classification and–where required–transforming this information into action [37–38]. These proposals fit well with central findings from the original fMRI study in which we found enhanced activation at checkpoints under high (vs low) contextual uncertainty. We interpreted these effects as an iterant evaluation of model information retrieved from working memory, pointing towards a strategic adaptation of predictive processing to contextual statistics [7]. Notably, the observed activation pattern included the temporo-parietal junction (TPJ), a hypothesised cortical source of the P3b [39]. Common ERP components and the similarities in functional microstates thus further illuminate the processing of CP and PE as highly informative events, suggesting that positions of potential and actual prediction errors are being exploited in a similar way.

It remains the key difference between checkpoints and prediction errors that only the latter violated cue-based predictions. Therefore, despite the similarities reported above, CP and PE will eventually be processed differently once consequences of the actual stimulus come into effect. Supporting our initial hypothesis, the mismatch signal for PE (vs CP) was reflected in an N400 component. N400 effects have typically been reported when words mismatched semantic expectations shaped by previous context information (e.g., [40]). Closely related to the present paradigm, centro-parietal N400 effects following incorrect (vs correct) solutions in arithmetic tasks (e.g., [41]) point towards a more general process independent of stimulus modality. Accordingly, Kutas & Federmeier [11] discuss the N400 as an index of conceptual representations which–when contextually induced predictions are violated–may need to be refined. Such adaptive processes are conceivably reflected by components occurring even later than the PE-related N400, e.g., ERPs related to subsequent digits ‘confirming’ the initially surprising stimulus. Future research could make use of later time frames to further distinguish prediction errors and checkpoints with regard to the respective consequences they entail.

To summarise, checkpoints are informative points in time which, despite a lack of unexpected input, show close functional similarities to canonical prediction errors. Our findings suggest that information from particular sequential positions, irrespective of the actual outcome, is used for evaluation and/or updating of internal models. Importantly, while sensory input at CP complied with the more likely expectation, their sequential positions were tagged by the statistical structure inherent in the stimulus stream. Previous fMRI results have shown CP to be exploited particularly in highly uncertain contexts, conceivably in order to solve ambiguity with regard to upcoming sensory information and efficiently adapt behaviour. Overall, the functional profile of checkpoints conceptually relates to *bottleneck states* [42–43] from the realm of hierarchical reinforcement learning. Bottleneck states form natural subgoals in hierarchical representations of behaviour [44–45]. For example, when trying to find the kitchen in a friend’s house, certain features like doors and stairways operate as bottlenecks informing the search [42]. Consequently, bottlenecks are conceptualised as transition points between larger sets of representational states. Similarly, on a more abstract level, the sequential positions of CP and PE mark informative transition points between predictable and non-predictable (random) states. Depending on whether or not the presented stimulus complied with cue-based expectations, checkpoints and prediction errors are supposedly used for model evaluation and updating, respectively.

### Implications for predictive processing

Combined ERP and microstate findings of the present study revealed considerable similarities between the representations of checkpoints and prediction errors. On a broader scale, this suggests overlapping roles of CP and PE in predictive processing. Given that error-based model updating has been established to be fundamental for associative learning [46], CP could similarly be used for model *evaluation*. Clearly, expectation-compliant information (as observed at checkpoints) does not call for corrective model updating. It seems unlikely, however, that potentially critical information extracted from CP would not be used to evaluate the validity of model statistics on-line. Particularly for the estimation of higher level statistics, the number of regular outcomes at critical time points is no less instructive than the number of prediction errors. Support for this proposition comes from earlier studies using digit sequences in abstract predictive processing. Kühn and Schubotz [6] found a distinct frontal correlate of regular, model-compliant events at sequential positions where statistically rare breaches of expectancy had previously been observed. As the actual sensory input neither violated model-based predictions nor called for behavioural adaptation, these frontal responses reflected increased weight of bottom-up signals driving potential model updating solely based on statistical regularities. Another study manipulated the requirement to either ignore or respond to two different expectation violations [47]. Again, violations that could be ignored (‘drifts’) did confirm the internal model, whereas violations that required a response (‘switches’) prompted corrective model updating. The pattern of brain activation suggested a two-step neural response to these events, starting with joint processing of stimulus discrimination followed by distinct correlates of behavioural responses prompted by the respective violation type.

In line with these previous findings, we suggest information from checkpoint and prediction error time points to be evaluated irrespective of the actual outcome (distinguishing both events from non-informative standard trials), especially under uncertainty. Successive model adaptation is induced only in case of unexpected stimuli (distinguishing PE from CP). As the temporal resolution of fMRI did not allow for the inclusion of standard trials in the original study, it remains an intriguing question for future research to determine how context (in)stability influences the expectation and processing of these informative events.

In addition to effects of context uncertainty, behavioural subgroup analyses suggested inter-individual differences in cue learning as a determining factor for CP/PE processing: The more strongly participants had learned the cue-length association, the more often they showed early responses at the end of a sequence. Depending on which sequence was observed, this response pattern had diverging implications on behavioural efficiency: In case of regular sequences, early releases during the last sequential digit showed how strong anticipation of the sequence ending spurred fast and efficient responses. Critically, however, the very same anticipation led some participants to erroneously respond at the ‘would-be’ end of extended sequences. One explanation could be that (overly) successful cue learning triggered a consistent prediction of sequence length (“Five digits after green”) irrespective of context-dependent violations. This way, information from checkpoints (in regular sequences) or prediction errors (in extended sequences) would not be exploited, as indicated by the negative correlation between event-specific surprise and offset latency. Overall, these results suggest that participants with increased knowledge of cueing information strongly (and sometimes falsely) relied on these initial cues, virtually disregarding potentially informative transition points during the sequence. In other words, excess reliance on cue information led to less attention being given to these transition points. More formalised accounts of predictive processing have postulated attention to control the involvement of prior expectations at different levels [48]. Specifically, attention is conceptualised as a means to increase the weight (or *gain*) of neural responses coding error signals, making them more eligible to drive learning and potential behavioural adjustments. Strict adherence to cue information conceivably impedes allocating attentional resources to CP/PE time points and, consequently, model adaptation. One promising direction for future studies would thus be to specifically vary training exposure between groups and assess the interplay of bottom-up and top-down dynamics underlying CP/PE processing.

### Limitations and future directions

The main aim of the present study was to exploit the temporal benefits of EEG for an extension of previous fMRI results. In order to warrant a high degree of comparability between the two studies, we chose a full replication of the experimental paradigm. As a consequence, it remains a limitation of the present study that half of the checkpoints required a response whereas the other half did not (for discussion, see [7]). To this end, one central direction for studies currently in preparation is to reduce the number of prediction error types, effectively ensuring equal behavioural relevance of all checkpoints. Furthermore, some caution is required when interpreting ERPs elicited by events of naturally varying presentation frequencies. Therefore, despite our best effort to limit noise in the EEG data, further research is needed to consolidate the functional characteristics of checkpoints and (less frequent) prediction errors.

There are several promising analyses beyond the scope of this paper which would not have been ideal for the current ERP epochs (-100 ms to 600 ms). Going forward, specifically re-epoching the data to include a longer pre-stimulus period would allow ERP and time-frequency analyses of anticipatory CP/PE processing as a function of uncertainty. Relatedly, the microstate analyses presented here motivate a more in-depth multivariate assessment of STD, CP, and PE representations, extending our understanding of similarities and differences between them. For example, STD trials should be reliably discriminable from CP and PE already during the pre-stimulus period, reflecting the anticipation of task-relevant information that can be obtained from the latter. Thus, representations of CP and PE should be similar during the pre-stimulus period but distinct during later periods reflecting actual outcome processing. Learning about the time course and potential uncertainty modulation of these comparisons will provide a more comprehensive account of the factors driving abstract prediction.

## Conclusion

Checkpoints are probabilistic, cue-compliant events informing predictive processing. Their functional profile closely resembles that of canonical prediction errors, indicating similar roles of the two event classes in abstract prediction. Both types of events presumably serve as reference points providing behaviourally relevant information, the central distinction being whether the respective outcome violates the internal model (PE) or not (CP). We suggest that despite the expected input observed at checkpoints, information at these particular positions is exploited on-line in order to adapt behaviour. Intriguing questions remain with regard to underlying network dynamics and their potential modulation as a function of uncertainty.

## Supporting information

S1 Fig ERP topographies for the three analyses detailed in the main text. Bold electrode positions indicate significant clusters from hypothesis-driven ROI analyses, asterisks indicate significant clusters from temporally unconstrained whole-brain analyses. Bold asterisked electrode positions indicate ROI-based clusters which remained significant after whole-brain correction using cluster mass permutation tests. PE = prediction errors, STD = standard trials, CP = checkpoints.
(TIFF)

S1 Table Detailed trial numbers for all conditions. Since low and high uncertainty blocks were each presented four times, trial numbers in parentheses show grand total number of presentations.
(DOCX)

S2 Table Total number of presentations for all events of interest and the minimum of trial numbers remaining after artefact rejection. PE = prediction errors, STD = standard trials, CP = checkpoints.
(DOCX)
