# Supplementary figures and images for: Being right matters: Model-compliant events in predictive processing
Source: PLoS One. 2019 Jun 13;14(6):e0218311. doi: 10.1371/journal.pone.0218311 (PMC6565358; doi:10.1371/journal.pone.0218311)

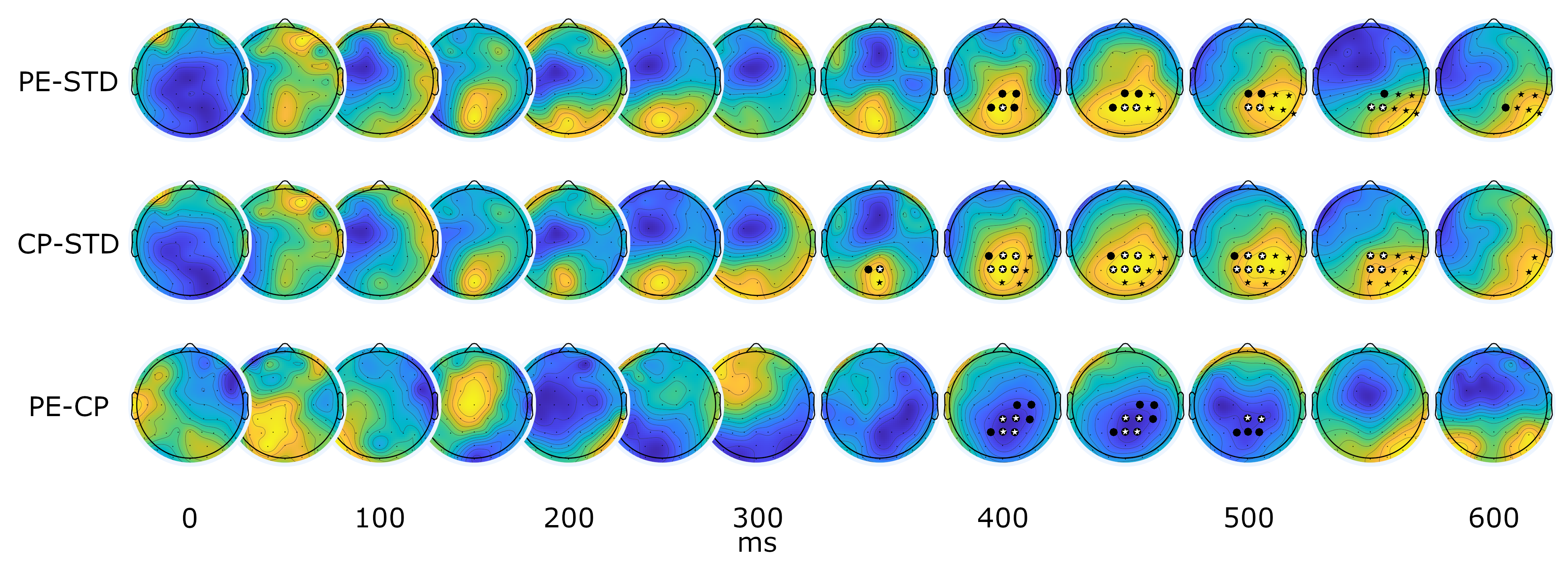

Supplement: S1 Fig — Bold electrode positions indicate significant clusters from hypothesis-driven ROI analyses, asterisks indicate significant clusters from temporally unconstrained whole-brain analyses. Bold asterisked electrode positions indicate ROI-based clusters which remained significant after whole-brain correction using cluster mass permutation tests. PE = prediction errors, STD = standard trials, CP = checkpoints. (TIFF) [file pone.0218311.s001.tiff]
