# Supplementary material for: Being right matters: Model-compliant events in predictive processing
Source: PLoS One. 2019 Jun 13;14(6):e0218311. doi: 10.1371/journal.pone.0218311 (PMC6565358; doi:10.1371/journal.pone.0218311)
Supplement: S1 Table — Since low and high uncertainty blocks were each presented four times, trial numbers in parentheses show grand total number of presentations. (DOCX) [file pone.0218311.s002.docx]

|  | Irreducible uncertainty | | |  |
| --- | --- | --- | --- | --- |
|  | low | high | total | |
| n_total_ | 40 (160) | 40 (160) | 320 | |
| terminated^1^ | 6 (24) | 12 (48) | 72 | |
| regular^2^ | 28 (112) | 16 (64) | 176 | |
| extended^3^ | 6 (24) | 12 (48) | 72 | |

^1^ Terminated and extended sequences were equally distributed between both sequence lengths (short, long)

^2^ Note that each regular sequence was sampled twice for checkpoints (see main text)

^3^ The fourth event of every long extended sequence was sampled as a sequential standard trial (STD)
