# Supplementary material for: Being right matters: Model-compliant events in predictive processing
Source: PLoS One. 2019 Jun 13;14(6):e0218311. doi: 10.1371/journal.pone.0218311 (PMC6565358; doi:10.1371/journal.pone.0218311)
Supplement: S2 Table — PE = prediction errors, STD = standard trials, CP = checkpoints. (DOCX) [file pone.0218311.s003.docx]

| **Event class** | **Uncertainty** | **presented trials (total)** | **minimum of trials after artefact rejection** |
| --- | --- | --- | --- |
| PE |  | 144 | 142 |
| CP | LOW | 224 | 222 |
|  | HIGH | 128 | 127 |
| STD |  | 36 | 35 |
